# Elevated MMP9 expression in breast cancer is a predictor of shorter patient survival

**DOI:** 10.1007/s10549-020-05670-x

**Published:** 2020-05-22

**Authors:** Chitra Joseph, Mansour Alsaleem, Nnamdi Orah, Pavan L. Narasimha, Islam M. Miligy, Sasagu Kurozumi, Ian O. Ellis, Nigel P. Mongan, Andrew R. Green, Emad A. Rakha

**Affiliations:** 1 Nottingham Breast Cancer Research Centre, School of Medicine, University of Nottingham, Nottingham, UK; 2 Faculty of Applied Medical Sciences, Onizah Community College, Qassim University, Qassim, Saudi Arabia; 3 Histopathology Department, Faculty of Medicine, Menoufia University, Shibin El Kom, Egypt; 4 Department of Pharmacology, Weill Cornell Medicine, New York, 10065 USA; 5 Faculty of Medicine and Health Sciences, University of Nottingham, Nottingham, LE12 5RD UK; 6 Division of Cancer and Stem Cells, Department of Histopathology, School of Medicine, The University of Nottingham and Nottingham University Hospitals NHS Trust, Nottingham City Hospital, Nottingham, NG5 1PB UK

**Keywords:** MMP9, Breast cancer, ECM remodelling, Prognosis

## Abstract

**Purpose:**

MMP9 is a matricellular protein associated with extracellular matrix (ECM) remodelling, that promotes tumour progression, and modulates the activity of cell adhesion molecules and cytokines. This study aims to assess the prognostic value of MMP9 and its association with cytoskeletal modulators in early-stage invasive breast cancer (BC).

**Methods:**

MMP9 expression was evaluated by immunohistochemistry using a well-characterised series of primary BC patients with long-term clinical follow-up. Association with clinicopathological factors, patient outcome and ECM remodelling BC-biomarkers were investigated. METABRIC dataset, BC-GenExMiner v4.0 and TCGA were used for the external validation of *MMP9* expression. GSEA gene enrichment analyses were used to evaluate *MMP9* associated pathways.

**Results:**

MMP9 immunopositivity was observed in the stroma and cytoplasm of BC cells. Elevated MMP9 protein levels were associated with high tumour grade, high Nottingham Prognostic Index, and hormonal receptor negativity. Elevated MMP9 protein expression correlated significantly with cytokeratin 17 (Ck17), Epidermal Growth Factor Receptor (EGFR), proliferation (Ki67) biomarkers, cell surface adhesion receptor (CD44) and cell division control protein 42 (CDC42). Cytoplasmic MMP9 expression was an independent prognostic factor associated with shorter BC-specific survival. In the external validation cohorts, *MMP9* expression was also associated with poor patients’ outcome. Transcriptomic analysis confirmed a positive association between *MMP9* and ECM remodelling biomarkers. GSEA analysis supports MMP9 association with ECM and cytoskeletal pathways.

**Conclusion:**

This study provides evidence for the prognostic value of MMP9 in BC. Further functional studies to decipher the role of MMP9 and its association with cytoskeletal modulators in BC progression are warranted.

**Electronic supplementary material:**

The online version of this article (10.1007/s10549-020-05670-x) contains supplementary material, which is available to authorized users.

## Introduction

Matrix metalloproteinases (MMPs) are a family of proteases that have multiple biological functions in cancer development and progression and are abundantly up regulated in breast cancer (BC). MMP9, also known as gelatinase B, plays an important role in extracellular matrix (ECM) remodelling, protein cleavage, and is associated with tumour invasion, metastasis and modulation of tumour microenvironment [1, 2]. MMP9 has the capability to degrade collagens, including Type IV collagen [3], which plays a role in basement membrane degradation promoting migration, invasion and metastases.

MMP9 is secreted as an inactive pro-enzyme and activation of latent MMP9 is the critical step in its regulation [4]. In vitro and in vivo experiments in human and experimental models of cancers reveal that the increased MMP9 expression is related to tumour progression [5, 6]. MMP9 expression is regulated by several molecular pathways such as extracellular signal-regulated kinase (ERK), mitogen-activated protein kinase (MAPK), and phosphoinositide-3-kinase–protein kinase (PI3K); pathways recognised to be altered in BC [7]. In BC, increased epidermal growth factor receptor (EGFR) expression, which is a poor prognostic marker, is implicated with up-regulation of MMP9 [8]. The regulation of the MMPs, particularly MMP9, by p53 has also been documented [9].

MMP9 is activated by the Cell Division Cycle 42 (CDC42), a Rho GTPase family member, which plays a major role in ECM adhesion and cytoskeletal organisation. Elevated CDC42 expression in BC dysregulates the epithelial architecture, which may initiate oncogenes. CDC42 gene silencing studies in BC xenografts showed that CDC42 knockdown decreased the tumour cell invasion and metastasis in vivo [10]. In addition, CDC42 activation stimulated trafficking of membrane-type 1 matrix metalloproteinase (MT1-MMP) in BC cells [11].

CD44 is a transmembrane glycoprotein cell surface adhesion receptor that promotes the secretion of active MMP9. *MMP9* gene silencing is shown to change the expression of CD44 and significantly decreases migration and invasion of tumour cells [12]. Increased *MMP9* mRNA expression was also observed in CD44^+^ BC cells compared to CD44^−^ cells. In vitro experiments showed that, inhibition of the CD44-MMP axis may provide therapeutic targets for reducing the tumour spread which further establishes a positive association between MMP9 and CD44 expression [10]. Thus, these studies support a role for CD44 in regulating MMP9 and is strongly associated with aggressively behaving tumours. In addition, MMP9 is part of the Rosetta poor-prognosis signature for BC [13] and in silico analysis of BC DNA microarray datasets also showed a positive association of MMP9 with poor outcomes [14]. For these reasons in this study we investigated the association between MMP9, cytoskeletal modulators, and clinicopathological factors of BC at the protein and mRNA levels using multiple well-characterised early-stage BC cohorts.

## Materials and methods

### Study cohort characteristics

This study obtained ethics approval by the North West–Greater Manchester Central Research Ethics Committee under the title; Nottingham Health Science Biobank (NHSB), reference number 15/NW/0685. All samples from Nottingham used in this study were pseudo-anonymised and collected prior to 2006 and stored in compliance with the UK Human Tissue Act. MMP9 protein expression was evaluated using a well-characterised cohort of early-stage (operable) primary invasive BC (*n* = 675) treated in Nottingham University Hospital NHS Trust as previously described [15]. Clinical and pathological data of patients (including hormone receptor status, histological tumour type, tumour grade, tumour size, lymph node status, Nottingham Prognostic Index and lymphovascular invasion (LVI)) were available. Tumour types were revisited and coded according to the recent WHO blue book [16]. BC-specific survival (BCSS) was maintained on a prospective basis. The expression of a large panel of BC progression/metastasis-related biomarkers, including the Ki67 [17], EGFR [18], CDC42 [19], CD44 [20], PIK3CA [21] and basal marker (cytokeratin 5/6 and 17) [22], was also studied. BC molecular subtypes based on the IHC profile of Oestrogen Receptor (ER), Progesterone Receptor (PR) and Human Epidermal Growth Factor 2 (HER2) were defined as previously described [23]: Luminal A: ER+/HER2− Low Proliferation (Ki67 < 10%), Luminal B: ER+/HER2− High Proliferation (Ki67 ≥ 10%) or ER +/HER2 + , HER2-positive class: HER2 + regardless of ER status, Triple Negative Breast Cancer (TNBC): exhibiting negative expression of ER, PR, and HER2.

The clinicopathological significance of *MMP9* mRNA expression, copy number alterations, differential gene expression analysis (DGE), and pathway analysis were assessed using the Molecular Taxonomy of Breast Cancer International Consortium (METABRIC) dataset (*n* = 1980 BC cases) [24]. External validation was performed using the Breast Cancer Gene-Expression Miner v4.0 (BC-GenExMiner v4.0) [15, 25] and The Cancer Genome Atlas (TCGA) [26] as previously described.

### Immunohistochemistry

Specificity of MMP9 antibody was validated by western blotting prior to immunohistochemistry. Cell lysates blots of HEK293 and MCF7 cell lines which were used as positive and negative controls respectively (obtained from the American Type Culture Collection; Rockville, MD, USA) were incubated with anti-MMP9 antibody (Rabbit monoclonal [EP1254], Abcam) at 1:800 dilution for overnight (4 °C) and bands were detected using fluorescent secondary antibodies at (1:15,000) (IR Dye 800CW donkey anti-rabbit and 680RD donkey anti-mouse, LI-COR Biosciences, UK). Mouse β-Actin (A5441, Sigma-Aldrich; Clone AC-15; Sigma, UK) at 1:5000 was used as a house-keeping protein. Blocking and visualisation were done as previously documented [27]. The specificity of the antibody was validated with a single specific band at the predicted molecular weight (70 kDa, Supplementary Fig. 1a).

Tumour samples were arrayed onto tissue microarrays (TMAs) as previously described [28]. Full-face BC tissue sections and TMAs were immunoassayed using Novolink Max Polymer Detection system (Leica, Newcastle, UK). In brief, 4 µm BC tissue sections were deparaffinised with xylene and rehydrated through 100% ethanol. Heat-induced (pH6) citrate antigen retrieval was performed and MMP9 antibody (1:100) was incubated for overnight at 4 °C. 3-3′ Diaminobenzidine tetrahydrochloride (Novolink DAB substrate buffer plus) was used as the chromogen. Slides were counterstained with Novolink haematoxylin for 6 min, dehydrated and cover slipped. Normal kidney tissue was used as a positive tissue control, whereas no primary antibody was used as a negative control.

TMA slides were digitally scanned at 20X magnification and viewed using NanoZoomer NDP viewer (Hamamatsu Photonics, Welwyn Garden City, UK). Both the percentage of staining and staining intensity of MMP9 cytoplasmic expression in invasive tumour cells and stromal cells were individually assessed to calculate the final histochemical score (H-score). Staining was double scored blindly by two researchers (NO and IMM) for 25% cores to assess inter-observer concordance. Inter-observer agreement was determined, and the interclass correlation coefficient was 0.86, indicating an excellent concordance between scorers. Discordant cases were re-scored by both observers and a final score was agreed.

### Statistical analysis

IBM SPSS 24.0 (SPSS IBM Corp, Chicago, IL, USA) software was used for statistical analysis and reported in line with REMARK guidelines [29]. The MMP9 H-score was dichotomised into high and negative/low expression using the median cut-point value. Chi-squared test was used to evaluate the association between MMP9 expression and the clinicopathological parameters.

Kaplan–Meier analysis with log-rank test for significance was performed to assess BCSS. Cox multivariate analysis was performed to test independence from standard prognostic factors in BC (nodal stage, tumour grade, tumour size, ER level of expression, and Ki67). A *p *value of < 0.05 was considered significant.

Gene expression was analysed in the subset of METABRIC patients for which MMP9 expression was available. DGE between A) low Vs high MMP9 cytoplasmic expression, and B) low Vs high MMP9 stromal expression were calculated using the Robina implementation of Edge-R statistical tool [30] and DGE with > twofold-change, and adjusted *p* values < 0.05 were considered significant. The DGE were examined using the online WebGestalt platform and adjusted *p* < 0.01 considered statistically significant [31, 32] and significantly enriched gene ontologies common for both cytoplasmic and stromal MMP9 protein expression. Furthermore, Venny 2.0 [33] was used to identify the overlapping DGEs common to both the cytoplasmic and stromal MMP9 protein expression.

## Results

### MMP9 protein expression in BC

**Fig. 1:**
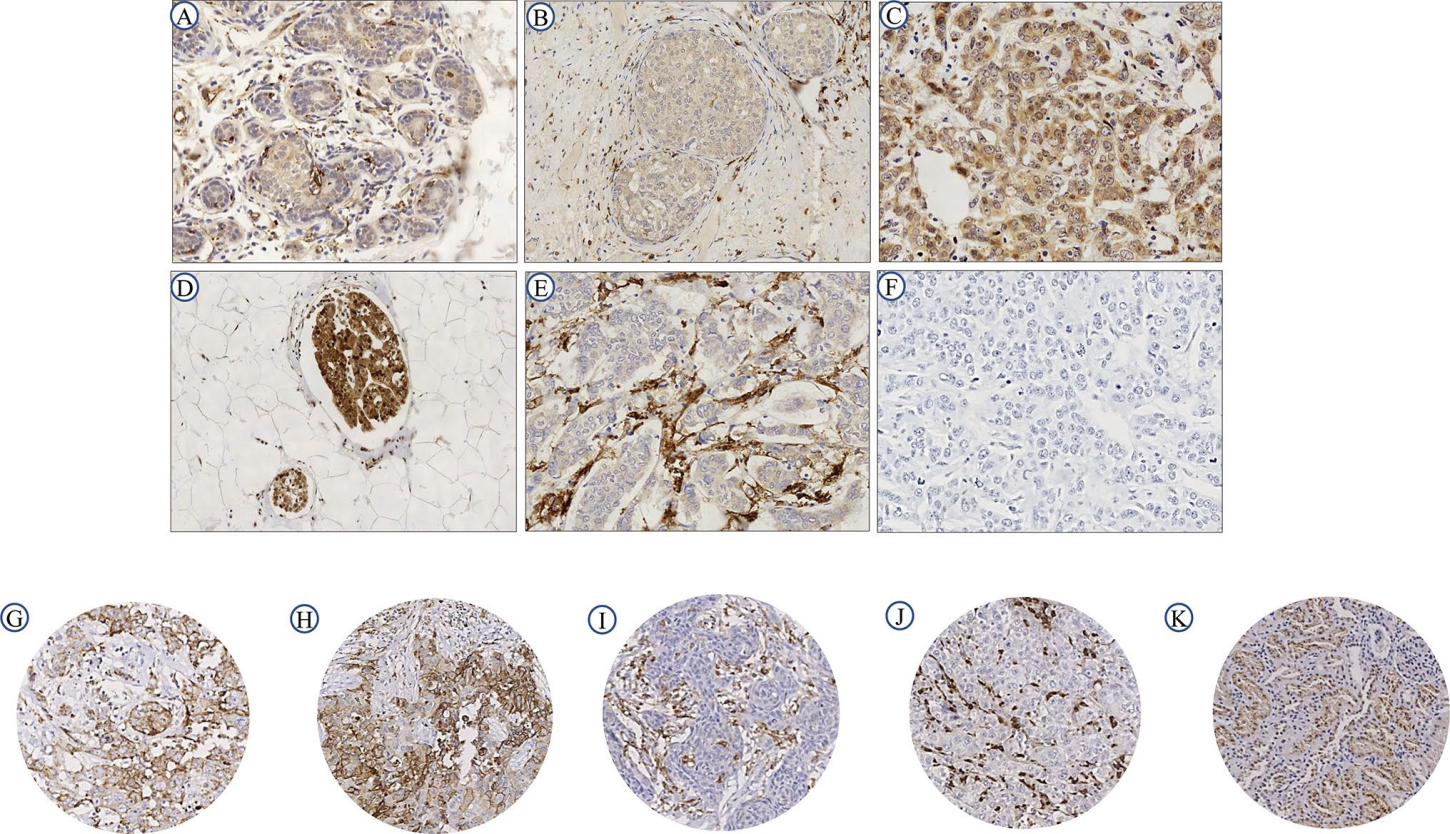
Immunohistochemical expression of MMP9 in BC. Morphological characteristics of MMP9 immunohistochemistry in Full-face breast cancer tissue (**a–f**). **a** Normal mammary gland and **b** DCIS showing absent or weak MMP9 staining. Showing high immunoreactivity in **c** invasive, and **d** LVI positive tumour samples. Showing high MMP9 stromal staining (**e**) and (**f**) no primary antibody control (negative control) in invasive breast carcinoma. All images are at 50 µm. MMP9 protein expression in breast cancer TMA cores (**g**) and (**h**) showing low and high cytoplasmic (C+) expression while, **i** and **j** showing low and high stromal (S+) expression, respectively. The normal kidney tissue (**k**) section used as positive control. TMA cores are 100 µm magnification

Full-face BC tissue (*n* = 10) sections were used to evaluate the pattern of MMP9 protein expression prior to staining of TMAs. This showed uniformly weak MMP9 expression in normal glandular epithelium (Fig. 1a) and ductal carcinoma in situ (DCIS; Fig. 1b). There was slightly increased immunoreactivity of MMP9 observed in the co-existing invasive BC cells (Fig. 1c), in the intravascular tumour cell emboli (Fig. 1d), stromal expression (Fig. 1e) and Fig. 1f showing No Primary Antibody Control. MMP9 cytoplasmic and stromal protein expression (C+/S+) showed a positive correlation with *MMP9* mRNA (Spearman’s coefficient 0.218; *p* = 0.027), this association was confirmed using TCGA data [34, 35] (Supplementary Fig. 1b).

On BC TMAs, a variable degree of MMP9 protein expression was observed (Fig. 1g–k). The H-scores of both cytoplasmic and stromal expressions did not follow a normal distribution. Therefore, for dichotomisation into negative/low and high expression, the median H-scores (0 and 50, respectively) were used. Out of 675 informative TMA cores, 71% showed negative/low expression in the cytoplasm (Fig. 1g) while 29% showed high expression (Fig. 1h). In the stroma 53% showed negative/low expression (Fig. 1i), while 47% showed high expression (Fig. 1j). Positive immunoreactivity was observed in human kidney sections (Fig. 1k). H-scores for cytoplasmic and stromal staining showed a positive correlation (Spearman’s coefficient 0.1; *p* = 0.020).

Elevated MMP9 cytoplasmic and stromal staining were associated with high tumour grade (*p* < 0.0001), poor Nottingham Prognostic Index (NPI) (*p* < 0.0001; Table 1), hormone receptor negativity (*p* < 0.01), among IHC subtypes associated with TNBC and HER2 + tumours (*p* = 0.002). High stromal MMP9 expression additionally showed association with LVI positivity (*p* = 0.046; Table 1). High MMP9 expression positively associated with proliferation marker Ki67 (*p* = 0.004*),* signalling pathway associated markers; EGFR (*p* = 0.024) and PIK3CA (*p* = 0.039), cytoskeleton remodelling markers; CDC42 and CD44 (*p* < 0.003 and *p* = 0.025, respectively). High cytoplasmic MMP9 showed positive association with basal cytokeratin CK17 (*p* = 0.001; Table 2) and revealed a low expression in lobular and special type tumours (*p* = 0.003).

**Table 1:** Associations between MMP9 protein expression and clinicopathological features in the whole Cohort

Parameters	MMP9 cytoplasmic (C+) expression			MMP9 stromal (S+) expression		
	Negative/low expression *n* (%)	High expression *n* (%)	*p* value (*χ*^2^)	Negative/low expression *n* (%)	High expression *n* (%)	*p* value (*χ*^2^)
Age at diagnosis (years)						
< 50	172 (70.5)	72 (29.5)	0.740 (0.110)	143 (58.0)	101 (42.0)	**0.019** (5.543)
≥ 50	309 (71.7)	122 (28.3)		212 (49.0)	219 (51.0)	
Histological grade						
1	76 (84.0)	14 (16.0)	** < 0.0001** (20.570)	61 (68.0)	29 (32.0)	** < 0.0001** (27.165)
2	177 (78.0)	51 (22.0)		141 (62.0)	87 (38.0)	
3	225 (64.0)	125 (36.0)		153 (44.0)	197 (56.0)	
Stage						
I	303 (74.0)	105 (26.0)	0.081 (6.743)	226 (55.0)	182 (46.0)	**0.012** (11.005)
II	138 (67.0)	69 (33.0)		106 (51.0)	101 (19.0)	
III	37 (70.0)	16 30.0)		23 (43.0)	30 (57.0)	
Tumour size						
< 2.0 cm	234 (74.0)	81 (26.0)	0.137 (2.206)	170 (54.0)	145 (46.0)	0.604 (1.269)
≥ 2.0 cm	246 (69.0)	110 (31.0)		185 (52.0)	171 (48.0)	
Histological type						
Ductal including NST*	406 (69.2)	181 (30.8)	**0.003** (11.586)	300 (51.1)	287 (48.9)	0.092 (4.88)
Lobular	48 (87.0)	7 (13.0)		34 (62.0)	21 (38.0)	
Special type	22 (88.0)	3 (12.0)		17 (68.0)	8 (32.0)	
IHC subtypes						
ER+/HER2-low proliferation	115 (81.0)	26 (19.0)	**0.002** (14.857)	85 (60.3)	56 (40.0)	**0.001** (15.879)
ER+/HER2− high proliferation	154 (72.0)	60 (28.0)		126 (59.0)	88 (41.0)	
Triple negative	80 (64.0)	45 (36.0)		51 (40.0)	74 (60.0)	
HER2 +	63 (62.0)	39 (38.2)		48 (47.0)	54 (53.0)	
Nottingham Prognostic Index						
GPG	155 (84.0)	29 (16.0)	** < 0.0001** (20.523)	117 (63.0)	67 (37.0)	**0.002** (12.257)
MPG	253 (68.0)	122 (32.0)		187 (49.0)	188 (51.0)	
PPG	72 (64.0)	40 (36.0)		51 (46.0)	61 (56.0)	
Lymphovascular invasion (LVI)						
Negative/probable	230 (73.0)	86 (27.0)	0.445 (0.584)	180 (57.0)	136 (43.0)	**0.031** (4.669)
Definite	157 (70.0)	68 (30.0)		107 (48.0)	118 (52.0)	

Significant *p* values are highlighted in bold; GPG; Good Prognostic Group; MPG: Moderate Prognostic Group; PPG: Poor Prognostic Group

* Medullary like carcinoma was renamed as Ductal NST carcinoma according to the recent WHO book 2019 and added to the ductal NST group

**Table 2:** Associations between MMP9 protein expression and other biomarkers in the breast cancer cohort

Parameters	MMP9 cytoplasmic (C+) expression			MMP9 stromal (S+) expression		
	Negative/low expression *n* (%)	High expression *n* (%)	*p* value (*χ*^2^)	Negative/low expression *n* (%)	High expression *n* (%)	*p* value (*χ*^2^)
Oestrogen (ER) status						
Negative	107 (59.0)	74 (41.0)	** < 0.0001** (18.402)	69 (38.0)	112 (62.0)	** < 0.0001** (20.194)
Positive	373 (76.0)	118 (24.0)		283 (58.0)	208 (42.0)	
Progesterone (PR) status						
Negative	182 (66.0)	95 (34.0)	**0.005** (7.860)	125 (45.0)	152 (55.0)	**0.002** (9.936
Positive	284 (76.0)	91 (24.0)		216 (58.0)	159 (42.0)	
Human epidermal growth factor receptor 2 (HER2)						
Negative	410 (74.0)	146 (26.0)	**0.009** (6.784)	302 (54.0)	254 (46.0)	0.150 (2.077)
Positive	63 (61.0)	40 (39.0)		48 (47.0)	55 (53.0)	
Epidermal Growth Factor Receptor (EGFR)						
Negative	385 (74.0)	137 (26.0)	**0.024** (5.069)	285 (55.0)	237 (45.0)	**0.016** (5.856)
Positive	85 (64.0)	48 (36.0)		57 (43.0)	76 (57.0)	
Phosphatidylinositol-4,5-bisphosphate 3-kinase, catalytic subunit alpha (PIK3CA)						
Negative	93 (79.0)	25 (21.0)	**0.052** (3.787)	72 (61.0)	46 (39.0)	**0.039** (4.271)
Positive	287 (70.0)	125 (30.0)		207 (50.0)	205 (50.0)	
Ki67						
Negative	156 (77.0)	47 (23.0)	**0.050** (3.858))	126 (62.0)	77 (38.0)	**0.004** (8.474)
Positive	222 (69.0)	100 31.0)		158 (49.0)	164 (51.0)	
Cytokeartin5/6 (CK5/6)						
Negative	324 (71.0)	127 (29.0)	0.080 (3.071)	241 (53.0)	210 (47.0)	0.099 (2.726)
Positive	57 (63.0)	34 (37.0)		40 (44.0)	51 (56.0)	
Cytokeartin17 (CK17)						
Negative	309 (73.0)	113 (27.0)	**0.001** (10.888)	225 (53.0)	197 (47.0)	0.395 (0.724)
Positive	42 (55.0)	35 (45.0)		37 (48.0)	40 (52.0)	
Cell division cycle 42 (CDC42)						
Negative	196 (76.0)	60 (24.0)	**0.003** (9.046)	138 (54.0)	118 (46.0)	0.892 (0.018)
Positive	126 (64.0)	72 (36.0)		108 (55.0)	90 (45.0)	
Cluster of differentiation 44 (CD44)						
Negative	109 (77.0)	32 (23.0)	**0.025** (5.027)	77 (55.0)	64 (45.0)	0.996 (0.001)
Positive	167 (67.0)	84 (33.0)		137 (55.0)	114 (45.0)	

Significant *p* values are highlighted in bold

**Fig. 2:**
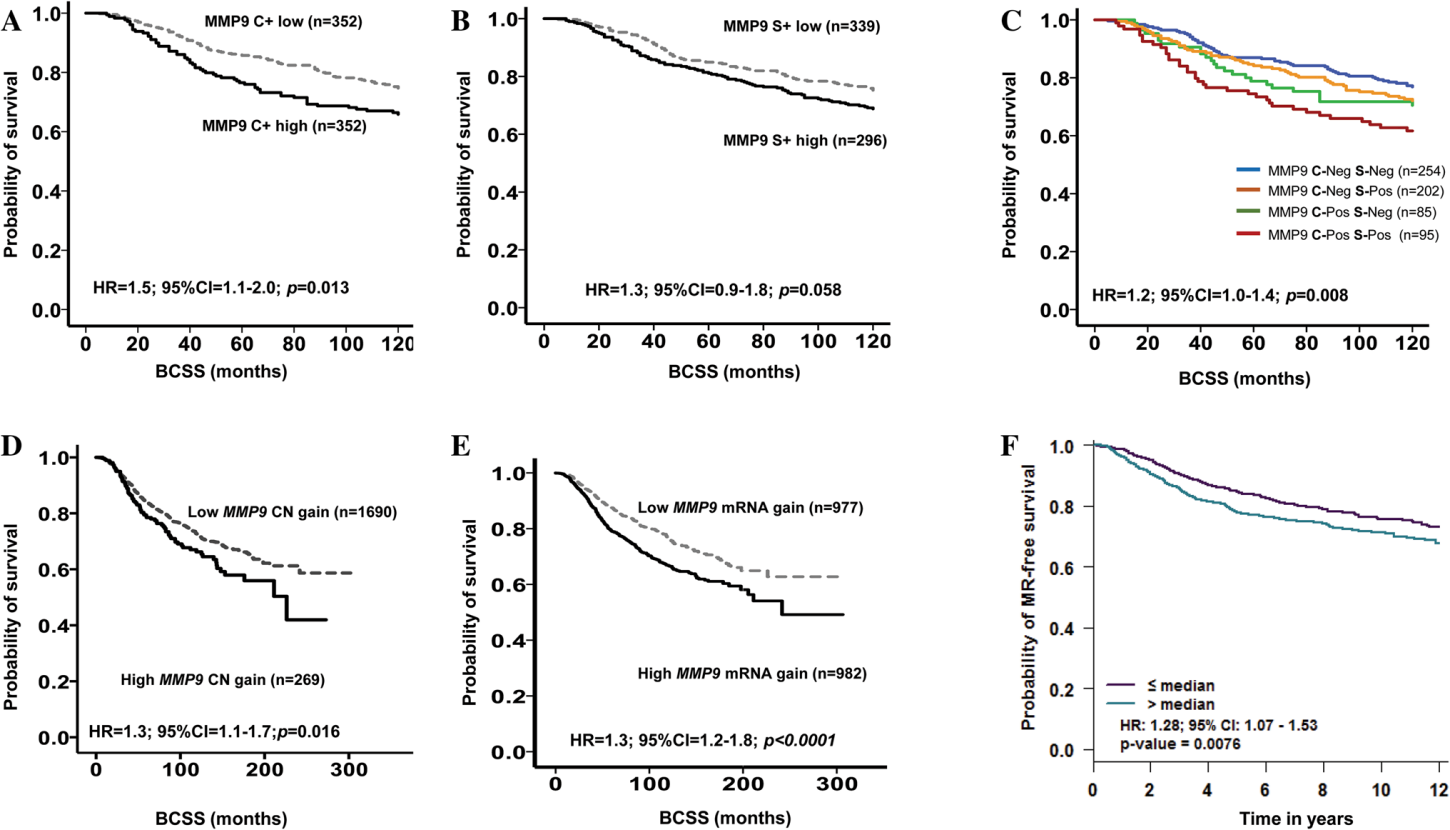
Kaplan–Meier plots of MMP9 expression and breast cancer patient outcome. At protein level **a** cytoplasmic MMP9, **b** stromal MMP9, **c** combined cytoplasmic and stromal MMP9 expression. Combined analysis of cytoplasmic and stromal MMP9 demonstrated that tumours with high cytoplasmic and stromal expression were associated with shorter BCSS. At transcriptomic level (**d**) METABRIC cohort *MMP9 gain*, **e** M*MP9 mRNA* and **f** Breast Cancer Gene-Expression Miner v4.0-Kaplan–Meier plots of *MMP9* gene expression. Outcome analysis revealed that high expression of *MMP9* was associated with shorter patient survival

BCSS of patients with tumours expressing high cytoplasmic MMP9 was significantly shorter than that of the negative/low expression subgroup (*p* = 0.013; HR = 1.5; 95%CI 1.1–2.0; Fig. 2a). Stromal MMP9 also showed a similar trend but did not reach significance (*p* = 0.058; HR = 1.3; 95%CI 0.9–1.8; Fig. 2b). Combined MMP9 cytoplasmic and stromal (CS) survival analysis demonstrated that tumours with high cytoplasmic and stromal expression were associated with poor prognosis (*p* = 0.008; HR = 1.2; 95%CI 1.0–1.4; Fig. 2c). In multivariate Cox regression analysis, cytoplasmic MMP9 expression on its own was an independent predictor of BCSS (*p* = 0.026; HR = 1.6; 95% CI 1.1–2.3; Table 3). There was no association between MMP9 protein and outcome in Luminal A and B, TNBC or HER2 + subgroups.

**Table 3:** Univariate and multivariate analysis of MMP9 (C+ & S+) expression compared with tumour stage, grade, size, Ki67 and ER status for breast cancer-specific survival

Variable	Breast cancer-specific survival					
	Univariate			Multivariate		
	HR	95%CI	*p* value	HR	95%CI	*p* value
Whole cohort						
Stage	2.7	1.8–3.2	** < 0.0001**	2.4	1.8–3.2	** < 0.0001**
Grade	2.7	2.4–3.3	** < 0.0001**	2.1	1.3- 3.3	**0.001**
Tumour size	2.1	1.8–2.5	** < 0.0001**	1.5	0.9–2.3	0.068
ER	1.0	0.9–1.1	** < 0.0001**	0.9	0.9–1.0	0.335
Ki67	2.6	2.1–3.3	** < 0.0001**	1.5	0.8–2.4	0.155
MMP9(C+)	1.5	1.1–2.0	**0.013**	1.6	1.1–2.3	**0.026**
MMP9 (S+)	1.3	1.0–1.8	0.060	1.2	0.8–1.8	0.305

Significant *p* values highlighted in bold

### MMP9 genomic profiling

Consistent with the results obtained for MMP9 protein expression, in the METABRIC and TCGA datasets, *MMP9* copy number gain (14.0%) and high mRNA expression (50.1%) was associated with negative ER and PR status, high histological grade, and poor NPI (all; *p* < 0.01; Table 4). *MMP9* copy number gain and high *MMP9* mRNA expression was associated with poor prognostic METABRIC Integrative clusters [24] such as 1, 5 and 9 (*p* < 0.0001). Associations between *MMP9* copy number alterations, *mRNA* expression and clinicopathological variables are summarised in Table 4. External validation of the pooled data using BC-GenExMiner v4.0 was in agreement where high *MMP9* mRNA expression was associated with ER, and PR negativity, high histological grade (all; *p* < 0.001) and poor NPI *(p* < 0.01). In PAM50 subtypes, high *MMP9* expression was associated with basal-like and HER2 + classes (*p* < 0.0001).

**Table 4:** Associations between *MMP9* copy number, *mRNA* expression and clinicopathological features in the METABRIC & TCGA cohorts

Parameters	METABRIC cohort							TCGA cohort		
	*MMP9 copy* number expression				*MMP9 mRNA* expression			*MMP9 mRNA expression*		
	Gain	Neutral	Loss	*p* value (χ2)	Negative/low Expression *n* (%)	High expression *n* (%)	*p* value (*χ*^2^)	Negative/low expression *n* (%)	High Expression *n* (%)	*p* value (χ2)
Histological grade										
1	8 (4.7)	162 (95.3)	0 (0.0)	**0.001** (23.12)	119 (70.0)	51 (30.0)	** < 0.00001** (124.85)	62 (69.7)	27 (30.3)	** < 0.00001** (29.23)
2	93 (12.1)	669 (86.9)	8 (1.0)		465 (60.4)	305 (39.6)		200 (53.3)	175 (46.7)	
3	158 (16.6)	789 (82.9)	5 (0.5)		352 (37.0)	600 (63.0)		141 (40.1)	211 (59.9)	
Stage										
I	48 (9.6)	451 (90.0)	2 (0.4)	0.226 (8.171)	257 (51.0)	244 (49.0)	0.296 (3.71)	75 (47.0)	84 (53.0)	**0.005** (10.51)
II	117 (14.2)	702 (85.1)	6 (0.7)		403 (49.0)	422 (51.0)		232 (47.0)	260 (53.0)	
III	19 (16.1)	98 (83.1)	1 (0.8)		49 (41.5)	69 (58.5)		115 (61.0)	75 (39.0)	
Tumour size										
˂ 2.0 cm	102 (11.9)	752 (87.6)	4 (0.5)	0.067 (5.42)	432 (51.0)	426 (50.0)	0.651 (0.21)	112 (46.9)	127 (53.1)	0.253 (1.31)
≥ 2.0 cm	167 (15.2)	925 (84.0)	9 (0.8)		543 (49.0)	558 (50.0)		315 (51.2)	300 (48.8)	
PAM50 sub type										
Luminal A	67 (9.3)	646 (90.0)	5 (0.7)	** < 0.00001** (78.27)	473 (65.9)	245 (34.1)	** < 0.00001** (267.44)	257 (56.6)	197 (43.4)	** < 0.0001** (36.57)
Luminal B	122 (25.0)	361 (74.0)	5 (1.0)		263 (53.9)	225 (46.1)		85 (55.0)	70 (45.0)	
Basal	33 (10.0)	295 (89.7)	1 (0.3)		54 (16.4)	275 (83.6)		23 (36.0)	41 (64.0)	
Her2	34 (14.2)	205 (85.4)	1 (0.4)		73 (30.4)	167 (69.6)		47 (31.0)	105 (69.0)	
ER										
Negative	142 (32.3)	295 (67.2)	2 (0.5)	**0.029** (8.49)	103 (23.5)	336 (76.5)	** < 0.00001** (161.47)	59 (31.9)	126 (68.1)	** < .00001** (30.89)
Positive	223 (14.9)	1265 (84.4)	10 (0.7)		868 (57.9)	630 (42.1)		352 (55.1)	287 (44.9)	
PR										
Negative	146 (15.5)	791(84.1)	3 (0.3)	**0.019** (8.02)	349 (37.0)	591 (63.0)	** < 0.00001** (113.01)	106 (39.0)	166 (61.0)	**0.0001** (18.53)
Positive	125 (12.0)	905 (87.0)	10 (1.0)		635 (61.0)	405 (39.0)		300 (54.9)	246 (45.1)	
HER2										
Negative	233 (13.4)	1488 (85.9)	12 (0.7)	0.626 (0.94)	906 (52.3)	827 (47.7)	** < 0.00001** (37.06)	284 (50.1)	283 (49.9)	0.179 (1.81)
Positive	38 (15.4)	208 (84.2)	1 (0.4)		78 (31.6)	169 (68.4)		58 (43.6)	75 (56.4)	
IC clusters										
** 1**	50 (36.0)	88 (63.3)	1 (0.7)	** < 0.00001** (140.98)	62 (44.6)	77(55.4)	** < 0.00001** (227.16)			
2	10 (13.9)	62 (86.1)	0 (0.0)		41 (56.9)	31(43.1)				
3	16 (5.5)	274 (94.5)	0 (0.0)		200 (69.0)	90 (31.0)				
4	19 (5.5)	324 (94.5)	0 (0.0)		141 (41.1)	202 (58.9)				
5	32 (16.8)	157 (82.6)	1(0.5)		62 (32.6)	128 (67.4)				
6	21 (24.7)	63 (74.1)	1 (1.2)		42 (49.4)	43 (50.6)				
7	30 (15.8)	156 (82.1)	4 (2.1)		118 (62.1)	72 (37.9)				
8	29 (9.7)	267 (89.3)	3 (1.0)		209 (69.9)	90 (30.1)				
9	37 (25.3)	107 (73.3)	2 (1.4)		67 (45.9)	79 (54.1)				
10	27 (11.9)	198 (87.6)	1(0.4)		42 (18.6)	184 (81.4)				
Nottingham Prognostic Index										
GPG	70 (10.3)	607 (89.3)	3 (0.4)	**0.006** (14.45))	419 (61.6)	261 (38.4)	** < 0.00001** (61.01)			
MPG	165 (15.0)	929 (84.4)	7 (0.6)		488 (44.3)	613 (55.7)				
PPG	36 (18.1)	160 (80.4)	3 (1.5)		77 (38.7)	122 (61.3)				

Significant *p* values are highlighted in bold

*GPG* Good Prognostic Group, *MPG* Moderate Prognostic Group, *PPG* Poor Prognostic Group

High *MMP9* mRNA showed positive association with other MMPs (*MMP1, MMP2, MMP7, MMP11, MMP14 and MMP15*; all *p* = 0.0001), collagens [*COL27A1*; (*p* = 0.008), *COL23A1* (*p* = 0.003) and *COL11A2*; (*p* = 0.045)], *TGFβ1* (*p* < 0.0001) and cytoskeletal remodelling biomarkers *CDC42* (*p* = 0.004; Table 5). These associations were confirmed using BC-GenExMiner v4.0 (Supplementary Fig. 2) via gene targeted analysis.

**Table 5:** Associations between *MMP9 mRNA* and ECM associated markers

Parameters	METABRIC cohort		
	Negative/low Expression *n* (%)	High Expression *n* (%)	*p* value (χ2)
*Matrix metallopeptidase 1 (MMP1)*			
Negative	820 (60.0)	559 (40.0)	** < 0.0001** (164.48)
Positive	169 (28.0)	432 (72.0)	
*Matrix metallopeptidase 2 (MMP2)*			
Negative	604 (55.0)	505 (45.0)	** < 0.0001** (20.548)
Positive	385 (44.0)	486 (56.0)	
*Matrix metallopeptidase 7(MMP7)*			
Negative	583 (59.0)	407 (41.0)	** < 0.0001** (63.291)
Positive	406 (41.0)	584 (59.0)	
*Matrix metallopeptidase 11 (MMP11)*			
Negative	551 (56.0)	439 (44.0)	** < 0.0001** (25.796)
Positive	438 (44.0)	552 (56.0)	
*Matrix metallopeptidase 14 (MMP14)*			
Negative	587 (54.0)	503 (46.0)	**0.0001** (14.782)
Positive	402 (45.0)	488 (55.0)	
*Matrix metallopeptidase 15 (MMP15)*			
Negative	671 (55.0)	544 (45.0)	** < 0.0001** (35.026)
Positive	318 (42.0)	447 (58.0)	
*Collagen Type XXVII Alpha 1 (COL27A1)*			
Negative	524 (53.0)	466 (47.0)	**0.008** (7.032)
Positive	465 (47.0)	525 (53.0)	
*Collagen Type XXIII Alpha 1 (COL23A1)*			
Negative	598 (53.0)	531 (47.0)	**0.003** (8.962)
Positive	391 (46.0)	456 (54.0)	
*Collagen Type XXI Alpha 2 (COL11A2)*			
Negative	576 (51.0)	534 (49.0)	**0.045** (4.019)
Positive	411 (47.0)	457 (53.0)	
*Transforming Growth Factor Beta 1(TGFBeta1)*			
Negative	672 (67.0)	338 (33.0)	** < 0.0001** (226.83)
Positive	317 (32.0)	653 (68.0)	
*Cell Division Cycle 42 (CDC42)*			
Negative	496 (53.0)	431 (47.0)	**0.004** (8.325)
Positive	495 (47.0)	558 (53.0)	
*Cell surface adhesion receptor (CD44)*			
Negative	498 (50.0)	502 (50.0)	0.893 (0.018)
Positive	491 (50.1)	489 (49.9)	

Significant *p* values are highlighted in bold

In the METABRIC cohort, *MMP9* copy number gain was associated with significantly shorter BCSS (*p* = 0.016; HR 1.3; 95%CI 1.1–1.7; Fig. 2d). Tumours expressing high *MMP9* mRNA expression showed significantly shorter BCSS than the low expression subgroup (*p* < 0.0001; HR = 1.5; 95%CI 1.2–1.8; Fig. 2e). Pooled *MMP9* gene expression data (*n* = 2071) in BC-GenExMiner v4.0 confirmed the association of high *MMP9* expression with poorer outcome (*p* = 0.007; HR = 1.3; 95%CI 1.1–1.5; Fig. 2f) and in agreement with protein expression results. In multivariate Cox regression analysis, *MMP9* mRNA was an independent predictor of BCSS (*p* = 0.048; HR = 1.3; 95% CI 1.0–1.6) independent of the standard prognostic parameters of BC including tumour size, histological grade, nodal stage, and proliferative fraction as assessed by Ki67.

### Genomic investigation and pathway analysis

**Fig. 3:**
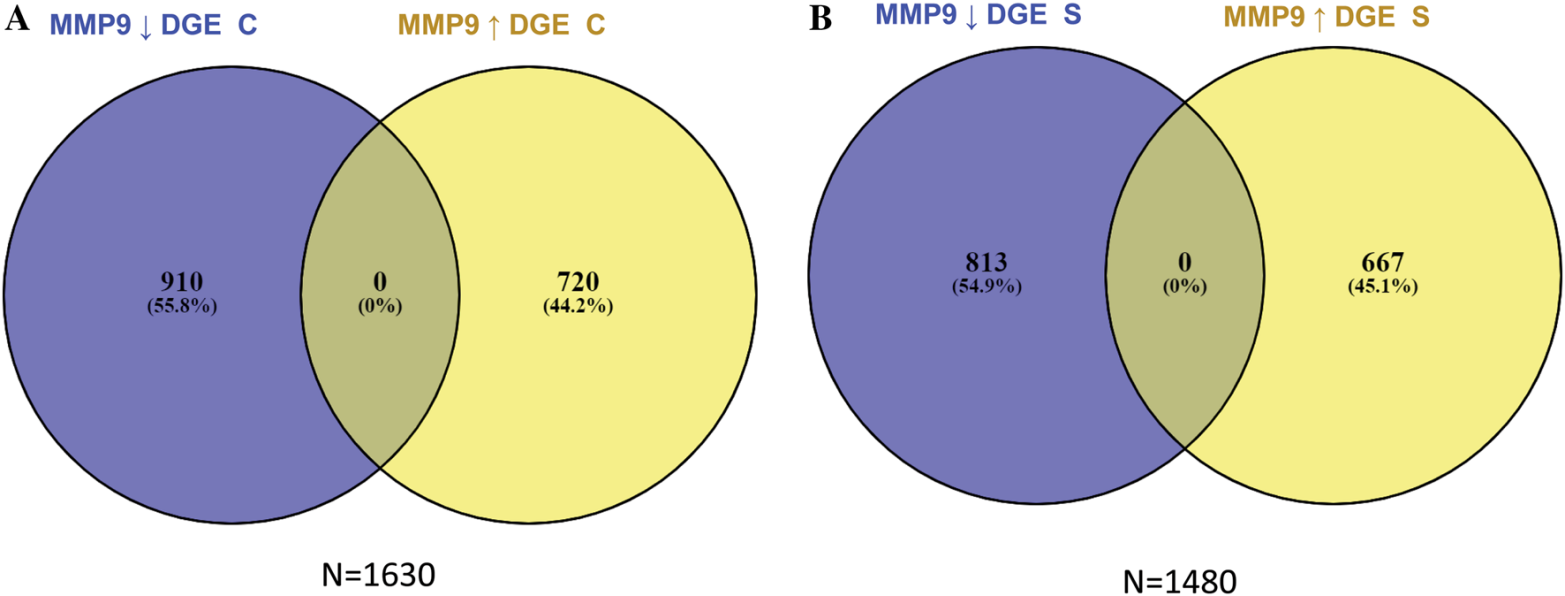
Differentially expressed genes associated with MMP9 cytoplasmic and stromal expression. **a** Cases depicting MMP9 cytoplasmic protein expression in tumour cells. **b** cases depicting f MMP9 protein expression the associated stroma of tumour cells. DGE: differentially expressed genes, C: cytoplasmic expression, S: stromal expression, (↓): down regulated genes, (↑) up regulated genes. (−) cases harbouring low expression of MMP9

**Fig. 4:**
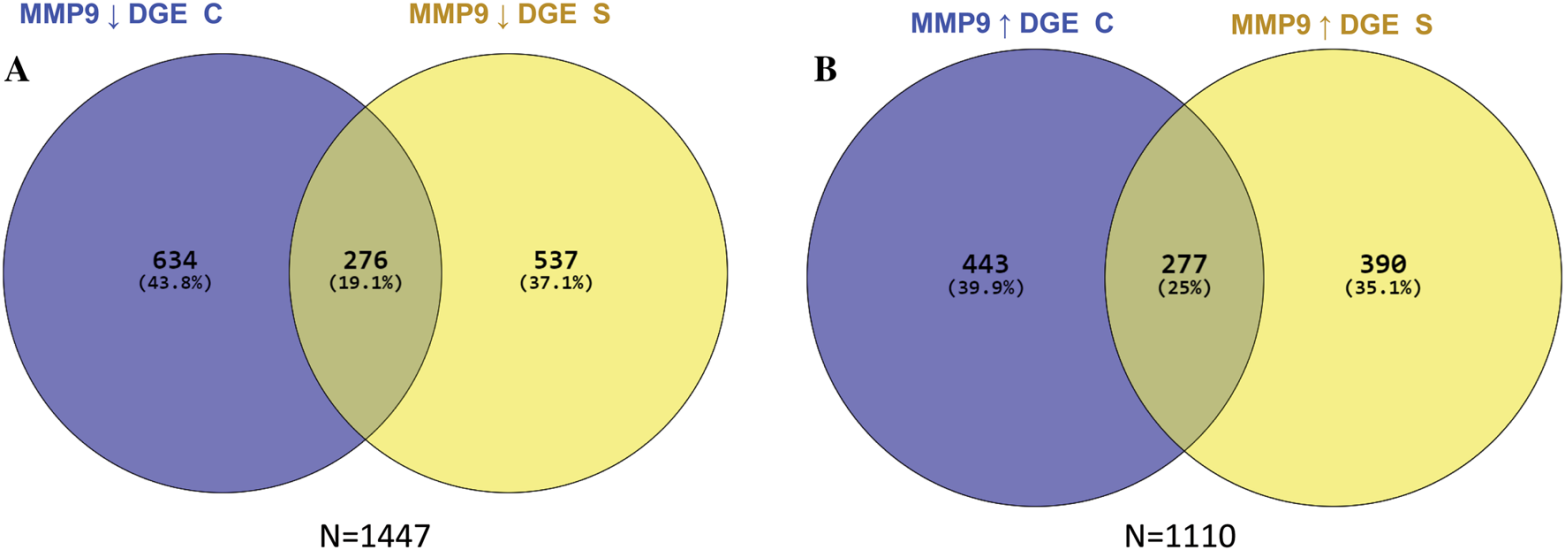
Overlapping differentially expressed genes associated with MMP9 cytoplasmic and stromal expression. Cases depicting: **a** down regulation of MMP9 protein expression on both the cytoplasmic and associated stroma of tumour cells. **b** Overlapping differentially expressed genes between cases depicting up-regulation of MMP9 protein expression on both the cytoplasm and associated stroma of tumour cells. DGE: differentially expressed genes, C: cytoplasmic expression, S: stromal expression, ↓: down regulated genes, ↑ up regulated genes

**Fig. 5:**
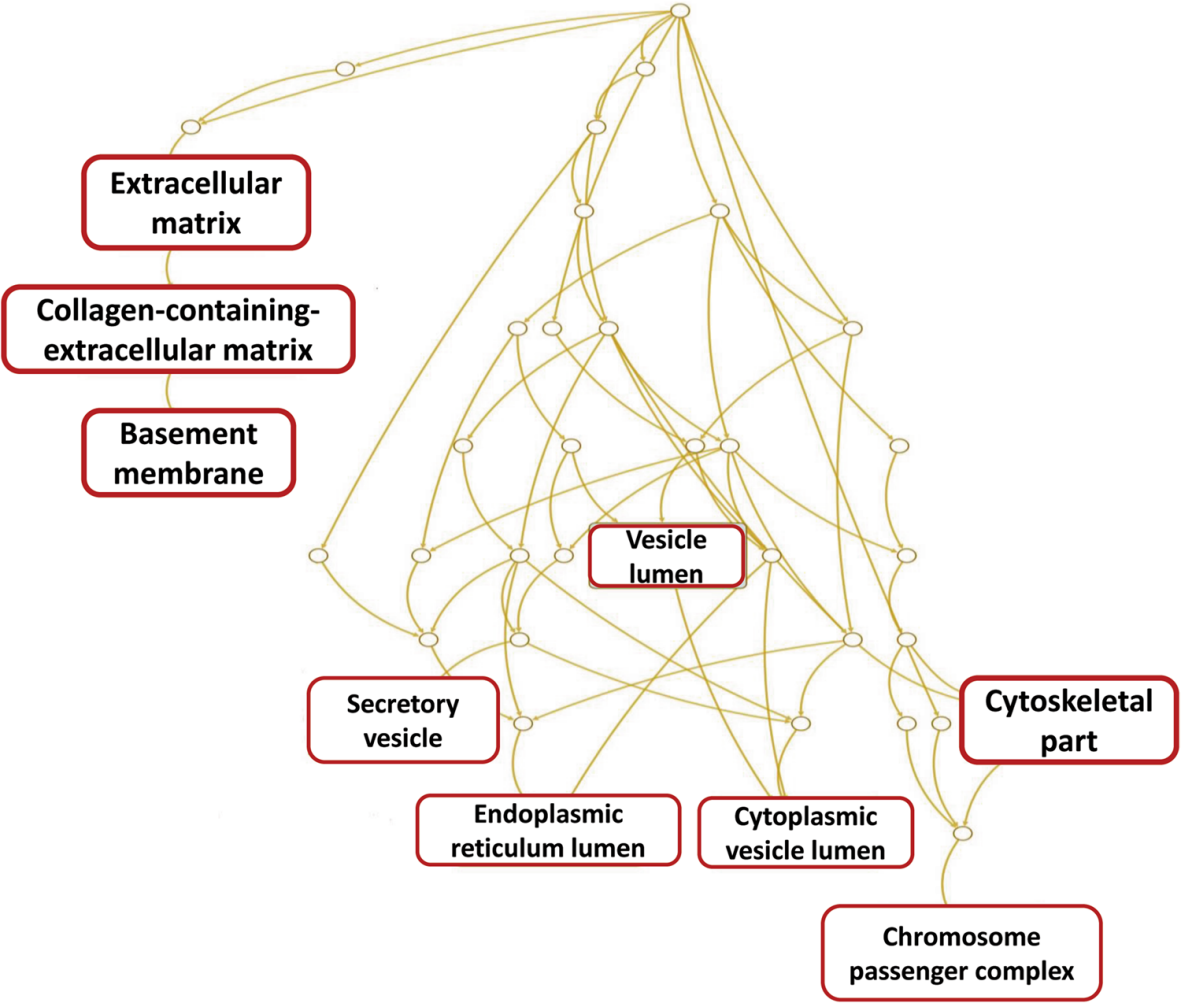
Pathway analysis for gene ontology significantly associated with cytoplasmic and stromal MMP9 protein expression. The gene panel were significantly associated with extracellular matrix related gene ontologies

To understand the molecular biology of MMP9 protein expression as an end point, the subset of the Nottingham series that was included in the METABRIC dataset (*n* = 113) were used for DGE analysis. The dichotomisation of cases into negative/low versus high groups was based on the dichotomisation of MMP9 protein expression. Cytoplasmic MMP9 expression displayed high expression in 36/113 cases (32%), while, stromal MMP9 showed high expression in 53/113 cases (47%). DGE analysis identified 1630 significantly differentially expressed genes associated with cytoplasmic MMP9 expression, with decreased cytoplasmic MMP9 expression displayed 720 upregulated and 910 downregulated genes, respectively. Stromal MMP9 showed 1480 differentially regulated genes, reduced stromal MMP9 expression was associated with 667 upregulated and 813 downregulated differentially expressed genes (Fig. 3). The overlapping DGEs between (A) low Vs high MMP9 cytoplasmic expression, and (B) low Vs high MMP9 stromal expression revealed 277 upregulated differentially expressed genes and 276 downregulated differentially expressed genes (Fig. 4). The common differentially expressed genes (*n* = 553) which were significantly associated with ECM related gene ontologies (Fig. 5, Table 6).

**Table 6:** Pathway analysis for gene ontology significantly associated with cytoplasmic and stromal MMP9 protein expression

Ontology	Name	Genes in Ontology	Observed	Expected		Enrichment	*p* value	False discovery rate	Genes
GO:0,031,012	Extracellular Matrix	496	37	10.065	3.6761		** < 0.0001**	< 0.0001	*CAN, AMTN, AZGP1, CDH2, CHAD, CILP, COCH, COL16A1, COL17A1, COL4A5*
GO:0,062,023	Collagen-Containing Extracellular Matrix	366	28	7.4269	3.7701		** < 0.0001**	< 0.0001	*CAN, AMTN, AZGP1, CDH2, CHAD, CILP, COCH, COL16A1, COL17A1, COL4A5*
GO:0,005,604	Basement Membrane	91	10	1.8466	5.454		** < 0.0001**	0.003	*ACAN, AMTN, COL17A1, COL4A5, COL9A2, FBLN1, LAD1, LAMA3, NTN4, THBS4*
GO:0,005,788	Endoplasmic Reticulum Lumen	306	19	6.8182	3.0599		** < 0.0001**	0.003	*AMTN, BCHE, CDH2, CESA, CHGB, COL16A1, COL17A1, COL4A5, COL9A2, ERAP2*
GO:0,044,430	Cytoskeletal Part	1620	57	32.873	1.7339		** < 0.0001**	0.004	*ACTA1, ACTG2, ACTR3, AK5, AURKA, AURKB, BIRC5, CASP14, CCNB1, CCNE1*
GO:0,031,983	Vesicle Lumen	337	19	6.8385	2.7784		** < 0.0001**	0.008	*CFD, FABP5, GGH, GLA, GNLY, LCN2, LYZ, PLFM4, ORM1, PNP*
GO:0,032,133	Chromosome Passenger Complex	5	3	0.10146	29.568		** < 0.0001**	0.009	*AURKA, AURKB, BIRC5*

Significant *p* values are highlighted in bold

## Discussion

Several studies have examined the roles of MMP9 in cancer development, progression, and its impact on patients’ survival and prognosis [2, 36–38]. Limited research however has been done to demonstrate the mRNA, copy number alterations and protein expression of MMP9 in BC and correlate the findings with clinicopathologic variables and cytoskeletal modulators in an extended cohort of BC patients. The cytoskeleton connects the cytoplasm and the plasma membrane and responds to external stimuli and signals. Cells that grow abnormally and acquire the ability to migrate and invade are the hallmark of metastatic cancers. Alteration of cytoskeletal structure is very important in cell invasion, migration, adhesion and change in morphology [39]. The degradation of ECM by MMP family members including MMP9 is believed to favour tumour growth, metastasis, invasion and cytoskeletal re-organisation [40]. Thus, the link between MMP9 and cytoskeletal modulators may have important clinical implications. The aim of this study was to determine whether elevated MMP9 expression at mRNA and protein level correlated with tumour grade, BC morphology, cytoskeletal modulators and patient outcome using a large clinical data set with long-term follow-up.

High MMP9 expression was associated with an increase in cell proliferation activity indicated by high expression of Ki67, which is associated with poor patient outcome [41]. Also, elevated levels of CK17 was associated with increased expression of MMP9. Breast tumours expressing CK17 and CK5/6 show high mortality rate which clearly implicate a role in tailoring treatment decisions [22]. These findings strengthen the putative role of MMP9 in tumour progression.

The EGFR/PIK3 signalling pathway plays important roles in tumour progression, and these pathways are reported to be frequently altered in BC [42]. We observed that high MMP9 expression was positively associated with EGFR and PIK3 signalling pathways. Elevated EGFR/PIK3 expression was associated with poor patient outcome in TNBC and Luminal B subtypes. Moreover, the PIK3/Akt pathway triggers MMP9 secretion and promotes cell invasion [43]. This implies that MMP9 could have a role in tumourigenic pathways.

MMP9 expression levels were positively associated with the expression of CDC42. CDC42 is a member of the Rho family of GTPases, which plays a role in many of the cellular processes that are associated with tumour progression, such as cell migration, proliferation, cytoskeletal control and vesicular trafficking [44]. A study conducted by Sipes and colleagues*;* revealed that, CDC42 deletion significantly reduced MMP9 activity. CDC42 is associated with formation of invadopodia, which can act as ‘guiding’ structures to pave the way for further cytoskeletal protrusions [45, 46]. Moreover, in vitro silencing of MMP9 decreased the migratory activity in Adenoid Cystic Carcinoma cells [47]. PIK3 and CDC42 mediate a positive feedback loop to regulate the tumour progression role [48]. Investigating the functional role of MMP9 in regulating the PIK3/CDC42 positive feedback loop in BC, might reveal a new role for MMP9 in the promotion of migration and invasion.

CD44 plays a major role in modulating migration/invasion processes during tumour advancement. Formation of CD44-MMP9 complex in prostate cancer promotes cellular motility and ECM invasion [49]. High MMP9 expression was positively associated with CD44 in our study. MMP9 acts as a processing enzyme for CD44 which promotes cell motility and, transcriptional knockdown of MMP9 inhibits this interaction [50]. Further studies will be needed to unravel the mechanisms by which MMP9 drives CD44 mediated invasion and tumour progression.

LVI is the presence of cancer cells in lymph vessels and is linked with a poor outcome in BC [51]. Daniele et al.*;* [52] showed that high levels of MMP9 expression was found in BC tumours with positive sentinel lymph nodes. The sentinel node is the proximal lymph node affected by metastatic cells since it is the first barrier receiving lymphatic drainage from the tumour. In the event of LVI, interactions between ECM and stromal non-tumoural cells induce migration, invasion and metastasis. Moreover, stromal fibroblast reported to secrete MMP9, which in turn may activate tumour cells [53]. Lymphatic networks within lymph nodes spread out before the onset of metastasis [54]. Although in our study we observed a weak association with stromal MMP9 and LVI, it warrants further investigations. Evaluation of stromal MMP9 expression may provide valuable information regarding the early LVI events.

The oncogenic expression of HER2 induces BC disease progression and invasiveness, which is hypothesised to increase MMP9 activity [53]. In this study, high *MMP9* mRNA expression was associated with poor prognostic parameters including higher tumour grade, ER-/PR-, HER 2 + tumours and TNBC tumours. MMP9 was also highly expressed in basal type tumours over luminal A and luminal B tumours. This is consistent with another study which found MMP9 as differentially expressed between molecular subsets of tumours and as a feature of TNBC and HER2 + BCs [55]. Increased MMP9 expression was associated with the poor prognostic category of the NPI. Hence, as observed in our study, MMP9 is a marker indicative of unfavourable prognosis in BC. Stromal invasion requires degradation of the basement membrane. MMP9 cleave the basement membrane type IV collagen and promote tumour invasion and metastasis [56, 57]. The correlation between high expression of *MMP9* and *collagen type XXVIIα1, XXIIIα1, XXIα2* may also induce basement degradation. Collagens, matricellular proteins and CDC42 at mRNA level showed strong positive association with *MMP9*. Results on pathway analysis confirmed the significant association with collagens, extracellular matrix and cytoskeletal part gene ontologies. This implies that MMP9 plays a role in tumourigenic pathways and could be a marker of poor prognosis in BC.

This study reveals that MMP9 at both proteomic and transcriptomic levels is associated with poor prognostic characteristics and short-term survival outcomes in BC. Cytoplasmic MMP9 expression on its own and combined cytoplasmic and stromal expression was predicative for shorter BCSS in the whole cohort, MMP9 did not show any association with patient outcome. Stromal cell genetic stability plays significant role in modulating the tumour microenvironment contributing to drug resistance and tumour relapse [58]. Moreover, expression of tumour markers in the stroma is found to be closely associated with tumour progression and patient outcome [59]. These findings suggest that MMP9 in both stromal cells and tumour cells might play an important role in the BC progression. In agreement with our study high expression of MMP9 was associated with poor patient survival [60]. Thus, the current study provides definitive evidence that MMP9 is an independent prognostic marker of poor short-term clinical outcome in primary BC and supports further mechanistic and translational studies to target MMP9.

## Electronic supplementary material

Below is the link to the electronic supplementary material.

Supplementary file1 (PDF 187 kb)

## Abbreviations

BC: Breast cancer
BCSS: BC-specific survival
CI: Confidence intervals
DGE: Differential gene expression
ER: Oestrogen
HR: Hazard ratio
HER2: Human epidermal growth factor receptor 2
METABRIC: Molecular taxonomy of breast cancer international consortium
NPI: Nottingham Prognostic Index
PR: Progesterone
TCGA: The cancer genome atlas
TNBC: Triple negative breast cancer
