# Supplementary material for: Elevated MMP9 expression in breast cancer is a predictor of shorter patient survival
Source: Breast Cancer Res Treat. 2020 May 22;182(2):267–82. doi: 10.1007/s10549-020-05670-x (PMC7297818; doi:10.1007/s10549-020-05670-x)
Supplement: Supplementary file 1 — Supplementary file1 (PDF 187 kb) [file 10549_2020_5670_MOESM1_ESM.pdf]

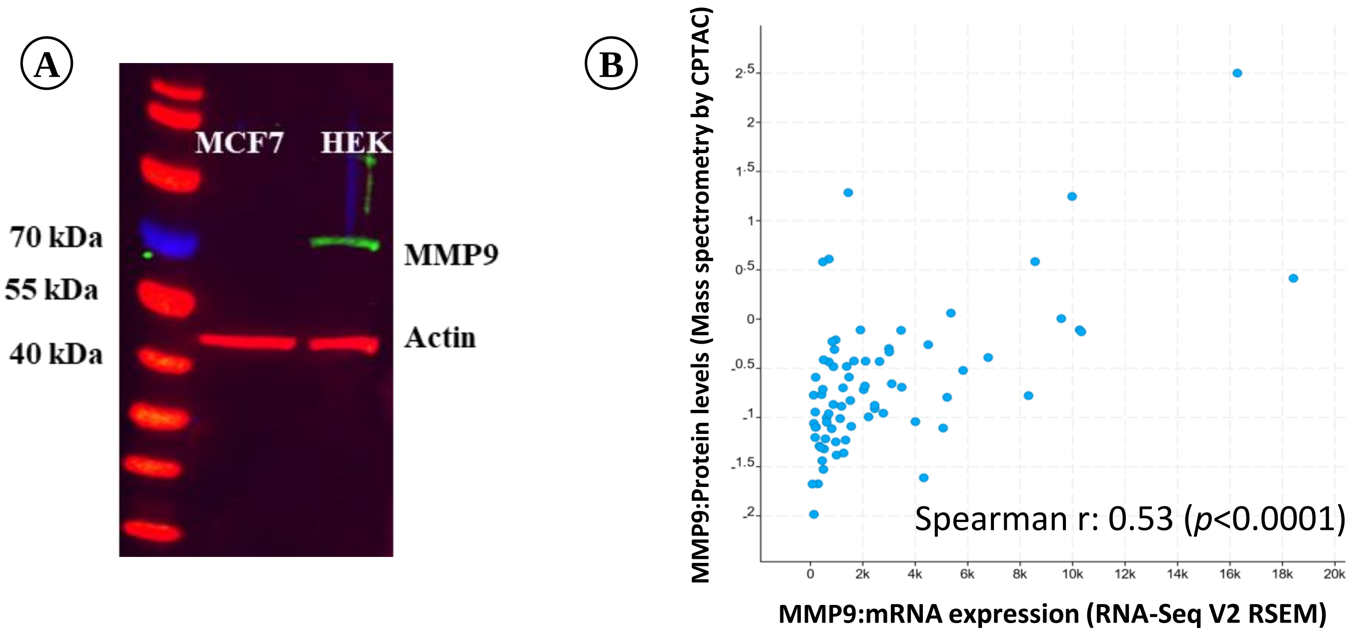

Supplementary Fig.1 Western blot and association between *MMP9* mRNA and protein in BC.
(**a**)Western blotting results for MMP9 protein expression in MCF7, and HEK cancer cell lines using rabbit monoclonal antibody against human MMP9. Green and red bands represent MMP9 and Beta-Actin (house-keeping) respectively. (**b**) Showing the positive association between MMP9 mRNA and protein (TCGA,[25,33])

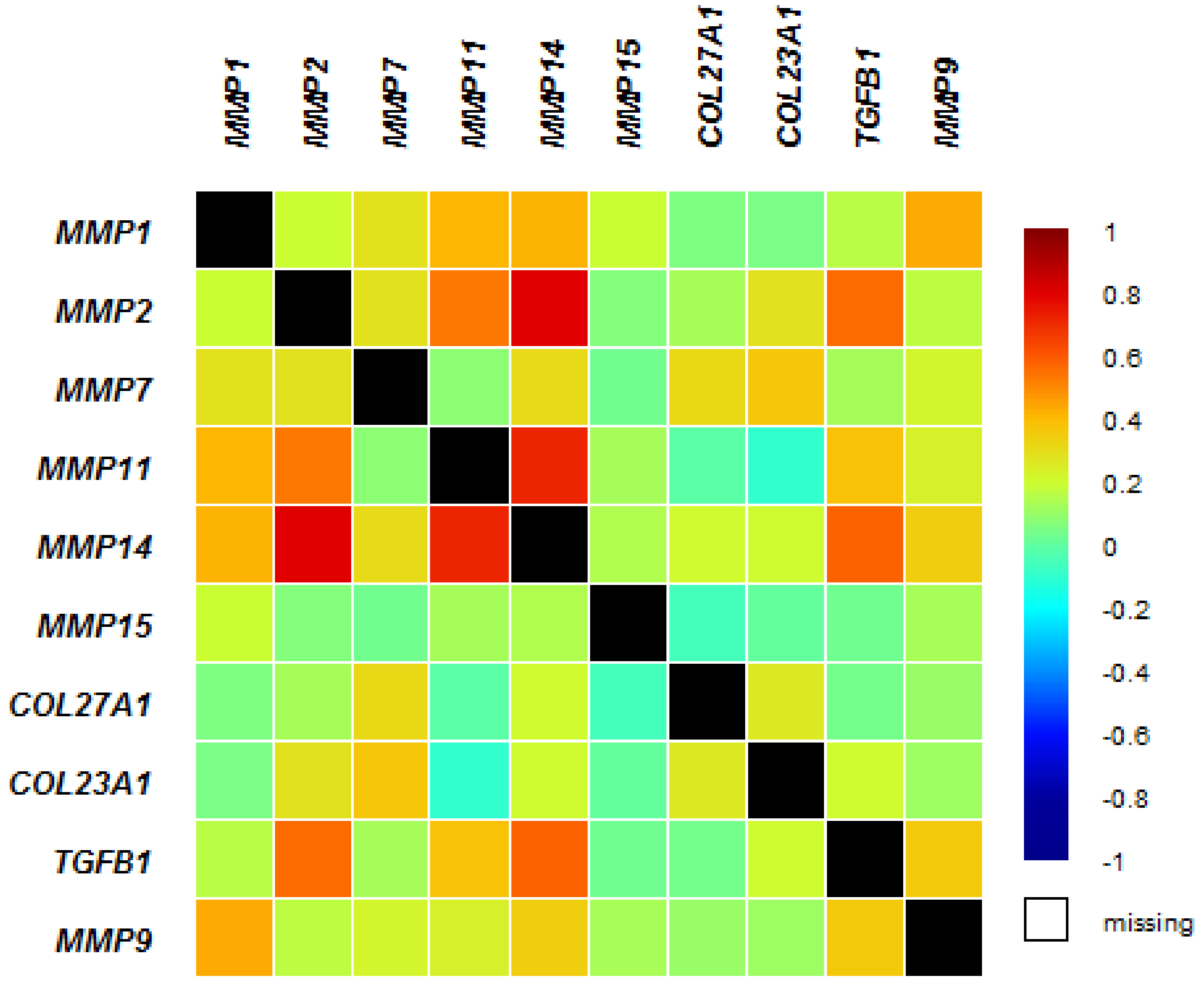

Supplementary Fig. 2 Gene correlation targeted analysis using Breast Cancer Gene-Expression Miner v4.3
Correlation map showed strong positive correlation between *MMP9* and other *MMPs*, *Collagens* and *TGFB1*
